# MOMSense: Metal-Oxide-Metal Elementary Glucose Sensor

**DOI:** 10.1038/s41598-019-41892-w

**Published:** 2019-04-02

**Authors:** Heba Abunahla, Baker Mohammad, Anas Alazzam, Maguy Abi Jaoude, Mahmoud Al-Qutayri, Sabina Abdul Hadi, Said F. Al-Sarawi

**Affiliations:** 1 Department of Electrical and Computer Engineering, Khalifa University of Science and Technology, Abu Dhabi, UAE; 2 Department of Mechanical Engineering, Khalifa University of Science and Technology, Abu Dhabi, UAE; 3 Department of Chemistry, Khalifa University of Science and Technology, Abu Dhabi, UAE; 4 Centre for Biomedical Engineering, School of Electrical and Electronic Engineering, The University of Adelaide, Adelaide, SA 5005 Australia

## Abstract

In this paper, we present a novel Pt/CuO/Pt metal-oxide-metal (MOM) glucose sensor. The devices are fabricated using a simple, low-cost standard photolithography process. The unique planar structure of the device provides a large electrochemically active surface area, which acts as a nonenzymatic reservoir for glucose oxidation. The sensor has a linear sensing range between 2.2 mM and 10 mM of glucose concentration, which covers the blood glucose levels for an adult human. The distinguishing property of this sensor is its ability to measure glucose at neutral pH conditions (i.e. pH = 7). Furthermore, the dilution step commonly needed for CuO-based nonenzymatic electrochemical sensors to achieve an alkaline medium, which is essential to perform redox reactions in the absence of glucose oxidase, is eliminated, resulting in a lower-cost and more compact device.

## Introduction

Diabetes, which is one of the most commonly diagnosed diseases at this time, is predicted to be the 7^th^ most deadly disease by 2030^1–3^. It occurs either due to a deficiency in the production of insulin or due to the inability of a body to utilize the available insulin^3–5^. Continuous monitoring of glucose levels in patients is essential to manage treatment and avoid critical complications or conditions such as cataracts, foot damage, and loss of vision^6–8^. Thus, the acquisition of a low-cost, miniature glucose measuring device can help patients and clinicians manage the disease.

**Figure 1:**
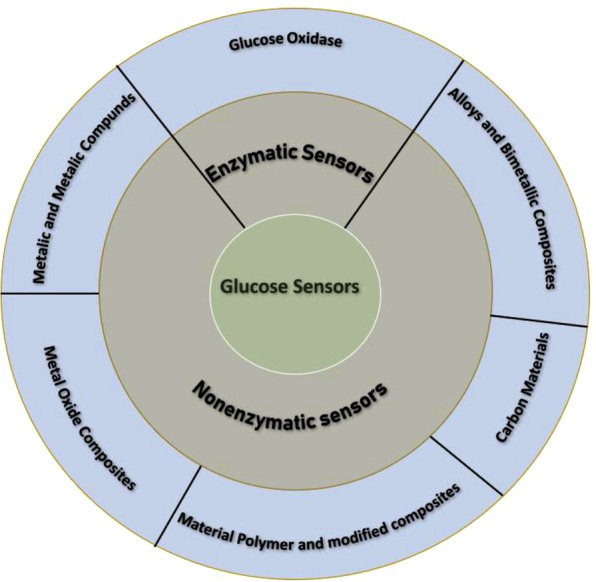
Types of enzymatic and nonenzymatic electrochemically active materials in glucose sensors.

Available glucose sensors can be divided into two main types, namely, enzymatic and nonenzymatic^9^, as illustrated in Fig. 1. Enzyme-based sensors are developed using glucose dehydrogenase (GDH) or glucose oxidase (GOx), which interacts with glucose molecules and results in an electrical response that is correlated to the concentration of glucose. Although enzymatic glucose sensors have been widely used and developed in the literature^3^, their short-term stability is affected by operating temperature, pH level, and humidity, in addition to the high fabrication cost. Both of these issues have encouraged the development of nonenzymatic glucose (NEG) sensors^10–15^.

NEG sensors^16–23^ allow glucose to be oxidized directly on the surface of the sensor, where the atoms at the surface act as the electrocatalysts, resulting in high stability, repeatability and cost-effective fabrication^24–26^. Two main models of glucose oxidation in NEG sensors have been proposed and explained in the literature. The first model was proposed by Pletcher^27^ and is known as the activated chemisorption model. In this model, the adsorption of the glucose molecule on the surface initiates glucose oxidation and enables the glucose molecule to form a bond with the atoms on the surface. On the other hand, the incipient hydrous oxide/adatom mediator (IHOAM) model proposed by Burke^28,29^ is associated with the active metal atoms on the electrode surface. These atoms have a low lattice stabilization and an enhanced reactivity, which aid the premono- layer oxidation step and facilitate glucose oxidation^29–31^.

Different materials have been used to develop NEG sensors, as shown in Fig. 1. This includes metals and metal compounds, alloys and bimetallic composites, metal oxide composites, polymer modified composites, and carbon materials. Although each material type has its own advantages and limitations, metals (e.g., Pt, Au, Ni, Cu, and Ag)^9^ and metal oxides (e.g., NiO, Cu_2_O, CuO, TiO_2_, ZnO, SnO_2_, MnO_2_, and Co_3_O_4_)^32–39^ have attracted the most attention recently for use as NEG sensors. This is due to the well-developed understanding of the electrocatalytic mechanism of glucose oxidation in such structures.

NiO has been commonly utilized in NEG sensors because of its catalytic properties, for which Ni(II) and Ni(III) are responsible for the required redox reaction^3^. To improve the stability and sensing performance reported by the available Ni-based sensors^34,40–43^, NiO-based hybrids have been investigated. Nanoparticle-assembled NiO nanosheets prepared using graphene oxide film, which is used as a template, have been recently explored for glucose sensing^44^. Although this system shows enhanced stability and selectivity over the available NiO-based sensors, a smaller linear detection range has been reported (0.001 mM – 0.4 mM). It is noteworthy that an alkaline medium (pH > 7) is needed for NiO/NiO hybrid-based sensors to accomplish the redox reaction^3,45^. As a cost-effective material with negligible toxicity, ZnO has been widely used for fabricating enzymatic glucose sensors^13,45^. Dar *et al*.^46^ were the first to report ZnO nanorods working as NEG sensors. The fabricated device was able to detect glucose at a neutral pH. However, the obtained linear range was very small (0.001 mM – 0.01 mM). To enhance the sensing performance, a combination of ZnO with NiO or CuO has been shown to be an effective approach to improve the overall catalytic performance of the fabricated sensor^45^. Nevertheless, the sensing medium must be diluted to achieve alkaline conditions and consequently attain the synergistic effects of the combined materials.

Among the metal oxide materials used, CuO is considered one of the best materials to be used in NEG sensing. This is due to its natural abundance, low production cost, high stability and appropriate redox potential. Equations (1) and (2) describe the dominant reactions taking place in CuO-based NEG sensors to allow electro-oxidation of glucose^9^. Furthermore, a substantial number of nonenzymatic CuO-based glucose sensors^47–57^ require a high pH (≥13) medium to perform glucose sensing.

1 $${\rm{CuO}}+{{\rm{OH}}}^{-}\to {\rm{CuOOH}}+{{\rm{e}}}^{-}$$

2 $${\rm{CuOOH}}+{{\rm{e}}}^{-}+{\rm{glucose}}\to {\rm{CuO}}+{{\rm{OH}}}^{-}+{\rm{glucose}}\,{\rm{acid}}$$

In this paper, we present a CuO-based glucose sensor structure, named MOMSense. The structure is capable of differentiating dissolved glucose levels in a liquid sample from as low as 2.2 mM to at least 10 mM when the liquid sample is at neutral pH. Achieving glucose sensing at a neutral pH is essential to improve the sensitivity of the detection unit. Tang *et al*.^58^ showed that performing sensing at a pH outside the neutral level affects the accuracy of the results, especially at diabetic glucose levels. Moreover, eliminating the dilution step needed for the sensing devices that work in an acidic or alkaline medium results in a cost-effective and compact device. The ability of the sensor to operate at a neutral pH facilitates its integration with other blood substance sensors. The ability to operate at a neutral pH is advantageous for the development of future lab-on-chip structures for real-time health monitoring.

**Figure 2:**
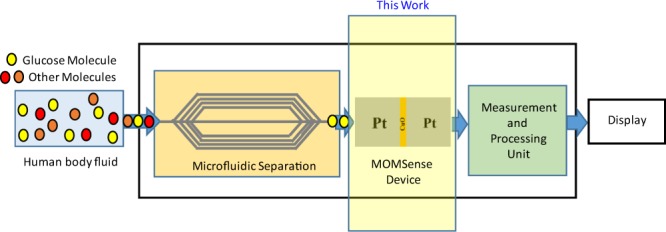
Envisioned block diagram of the lab-on-chip system for glucose sensing.

As shown in Fig. 2, MOMSense can be integrated into a microfluidic platform that serves as a miniature lab-on-chip^59–62^. The selective sample preparation and preconcentration steps enhance the sensitivity of the detection method. The improved selectivity starts by using a human fluid that is fed to the sensor through a microfluidic channel, where glucose molecules are extracted using a suitable separation technique. After this, separated fluid samples with glucose molecules are processed by the MOMSense device. The electrical response is measured and analyzed by the measurement and processing units to calculate the corresponding glucose level. Electrochemical detection integrated with a microfluidic paper-based analytical device (µPAD) is well-studied in literature and it is shown to play a significant role in glucose sensing due to its low cost, high sensitivity and selectivity, minimal sample preparation and short response time^63^. The microfluidic separation suggested in Fig. 2 is in line with the glucose sensing device proposed in^64^. In contrast, in this framework, the µPAD allows detection of low glucose molecules levels by pushing these molecules to the surface of the MOMSense through utilizing the capillary action of the µPAD structure. As a result, the current passing through the device will change as function of glucose concentration in the sample.

**Figure 3:**
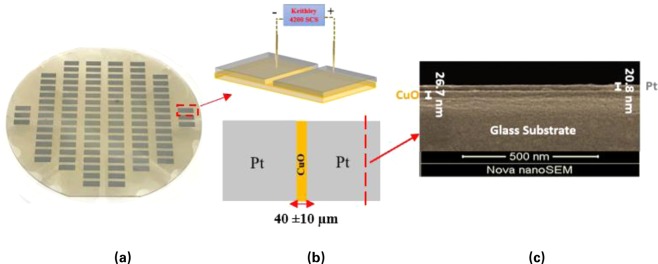
(**a**) A macrograph of the fabricated MOMSense devices on a glass wafer. (**b**) Device schematic to show the planar Pt/CuO/Pt structure. (**c**) Scanning electron microphotograph of a physical MOMSense device cross-sectional view under secondary electron mode (accelerating voltage, 5 kV; magnification, 140 453x; working distance, 5 mm).

The MOMSense device presented in this work is fabricated in a planar structure and can be mass produced using a wafer-style fabrication process, as shown in Fig. 3(a). Each device consists of a CuO layer and one pair of first and second Pt electrodes arranged on the oxide and separated by a gap containing the CuO layer, as shown in Fig. 3(b). The CuO surface extends around and below the metal electrodes and rests on a substrate layer, which can be any suitable inert structural layer, such as, but not limited to, glass. Figure 3(c) presents a scanning electron microphotograph of the device cross-sectional view, which shows a CuO thickness of 26.7 nm with another 20.8 nm Pt layer on a glass substrate.

## Results

### MOMSense Glucose Test

**Figure 4:**
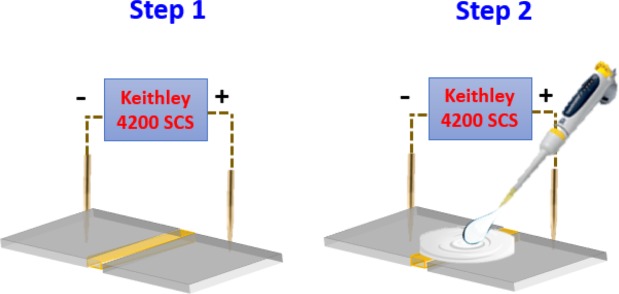
The two steps to perform the glucose test. Step 1 involves application of constant voltage bias (i.e. 1 V). Step 2: the addition of the liquid sample to touch both Pt electrodes and the CuO surface.

For each measurement, an unused device is selected randomly from the same wafer to investigate the sensing ability of MOMSense devices for the following glucose concentrations: 3.9 mM, 5.6 mM and 7.8 mM. The two measurement steps used in performing these tests are illustrated in Fig. 4.

Step 1: A dc voltage of 1 V is applied across the MOMSense sensors, and this voltage is the minimum working voltage for the sensor. (*i*) The resulting current level passing through the device is recorded. (*ii*) Next, the electrical stability of the device is checked in the absence of glucose.

Step 2: Under the same dc value, a 2 µl drop of glucose solution is added on the top of the sensor. This solution covers the oxide area and is simultaneously allowed to touch both electrodes.

**Figure 5:**
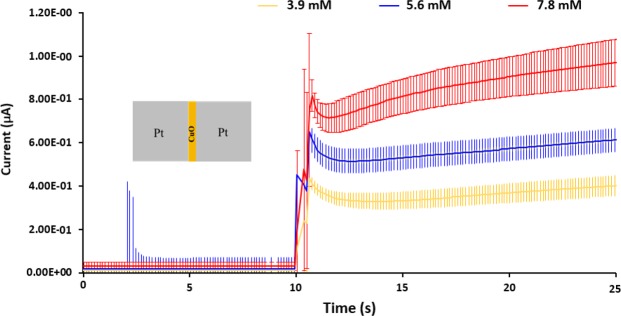
A chart illustrating the measured current across the fabricated MOMSense devices over time when subjected to an applied voltage of 1 V and when supplied directly with a glucose-containing sample of known glucose concentrations of 3.9 mM, 5.6 mM, and 7.8 mM at *t* = 10 s. These concentrations span low, medium, and high blood glucose levels for an adult human. For each glucose concentration, the illustrated data represent the average result obtained from seven fresh identical devices, with error bars for the measured current variation over time.

This testing mechanism follows the well-reported amperometric glucose sensing approach detailed in^15^, which mainly involves the application of a constant bias potential, followed by an electric current measurement. This current is linearly related to the glucose concentration. As presented in Fig. 5, MOMSense devices show an instantaneous response at *t* = 10 s, which is the time when the glucose solution is applied to the device surface. It is clear from these plots that the measured current level after addition of the solution depends on the glucose concentration. Despite the fact that each measurement is conducted across seven separate devices with different concentrations, the error bars for the variation in the measured average currents are statistically significant. Such variation in responses is expected due to the variation associated with the patch device fabrication. The error bars can be significantly reduced by careful optimisation of the patch fabrication process.

**Figure 6:**
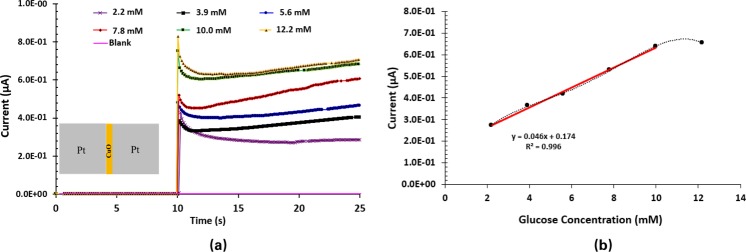
(**a**) A chart illustrating the measured current across MOMSense devices over time while 1 V is applied across the electrode and the glucose-containing sample. The results are shown for concentrations of 2.2 mM, 3.9 mM, 5.6 mM, 7.8 mM, 10.0 mM and 12.2 mM. These concentrations represent extremely low, low, medium, high and extremely high blood glucose levels for an adult human. As shown, the detected currents for each sample increase over time according to glucose concentration. Each concentration sample is tested on identical fresh devices. (**b**) A chart illustrating current data obtained with respect to Fig. 6(**a**) at a set time point of 18 s as a function of glucose concentration. It is clear that the response of the MOMSense devices saturates at a glucose concentration of 10.0 mM (180 mg/dl).

After confirming the repeatability, reproducibility and stability of MOMSense devices, the study is expanded to determine their linear range. This is achieved by testing a set of fresh devices using the following glucose concentrations: 2.2 mM, 3.9 mM, 5.6 mM, 7.8 mM, 10.0 mM and 12.2 mM. As presented in Fig. 6(a), MOMSense devices show instantaneous responses at *t* = 10 s, which is the time when the glucose solution is applied to the device. The current level for each concentration is relatively stable after one second of glucose application. The current value is read at *t* = 18 s and plotted versus the corresponding glucose concentration, as presented in Fig. 6(b). This set time point is selected because it provides the best linear fitting at the shortest time. The sensor has a linear characteristic between 2.2 mM and 10.0 mM, where the measured current consistently increases with the increase in glucose concentration. Moreover, it can be observed that the device sensitivity saturates at glucose concentrations above 10 mM (180 mg/dl). This is due to the high dependency of glucose adsorption on the available sensor surface area. Anion competition limits the extent of glucose oxidation, and therefore, the linearity of the oxidation current to the glucose concentration substantially degrades when the sensor surface is saturated^9,15,65,66^. It is clear that the empirical equation provided in Fig. 6(b) shows a nonzero passing model, which means that MOMSense devices have a different regime for lower concentrations.

Table 1 summarizes the CuO-based NEG sensors available in the literature. It is clear that MOMSense devices exhibit a wide linear range and high sensitivity at a neutral pH. The concept of an integrated lab-on-chip separation and detection platform presented in Fig. 2 would facilitate employing excessively corrosive environments to increase the sensitivity and maintain the chemical stability of the device.

**Table 1:** Summary of most recent nonenzymatic CuO-based glucose sensors with their sensing characteristics as provided in the relevant references.

Electrocatalyst	Linear range (mM)	Detection Limit (mM)	Sensitivity (μA mM^−1^ cm^−2^)	pH	Ref.
CuO nanosheets	0.5–10	1 × 10^−4^	520	13	^47^
CuO nanospheres	0–2.56	1 × 10^−3^	405	13	^48^
CuO nanoflowers	0–5	1.71 × 10^−3^	2657	13	^49^
Carnation-like CuO Hierarchical Nanostructures	0–5.5	98 × 10^−6^	3150	13.2	^50^
Flower-like CuO hierarchical nanostructures	4.5 × 10^−2^–1.3 × 10^−1^	6.87 × 10^−3^	1710	13	^51^
CuO nanorods	0–5	220 × 10^−6^	1834	14	^52^
Sandwich-structured CuO	0–3.2	1 × 10^−3^	5343	13	^53^
Cu/Cu2O/CuO ternary composite hollow spheres	0–0.1	0.39 × 10^−3^	8726	13	^54^
CeO2@CuO core shell nanostructure	1–8.9	0.019 × 10^−3^	3319	13	^55^
Nanocomposites of CuO and single-wall carbon nanotubes	5 × 10^−5^–1.8	50 × 10^−6^	1610	13	^56^
CuO nanoparticles	0.21 × 10^−3^–12	0.21 × 10^−3^	700	13	^57^
Pt/CuO/Pt metal-oxide-metal	2.2–10	1.42	2921	7	This work

**Figure 7:**
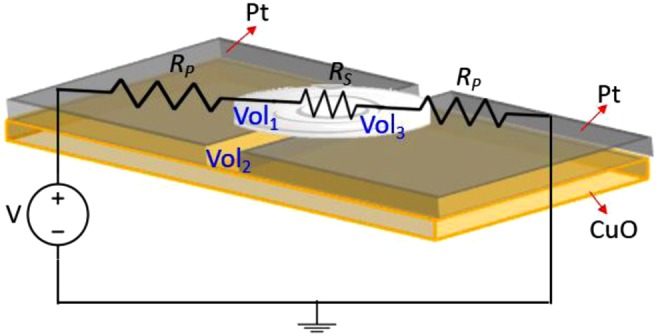
Circuit diagram of the fabricated MOMSense when a voltage source, V, is applied across the two electrodes and a glucose sample applied. *R*_*P*_ is the electrodes resistance, while *R*_*S*_ is the modified resistance of the CuO due to the sample application. This figure shows the three volumes that can affect the electrochemical reaction. These are Vol_1_ and Vol_3_, which refer to the Glucose and Pt volume, and Vol_2_ refers to the Glucose, Pt and CuO volume.

Figure 7 shows the equivalent circuit diagram of a MOMSense device, where

- resistor *R*_*P*_ represents the resistance of the Pt electrodes; this resistance is not affected by the added glucose, as the electrical current always passes through the conducting metal;
- resistor *R*_*S*_ is the CuO interface resistance, which is affected by the added glucose solution, and its value depends on the following electrochemical reactions;

- glucose and Pt are in volumes Vol_1_ and Vol_3_; and
- glucose, Pt and CuO are in volume Vol_2_.

To identify the roles of the Pt electrodes and the oxide material (CuO) used in MOMSense devices, two different systems, Pt/glass/Pt and Cu/CuO/Cu, are fabricated, and their glucose sensing abilities are tested.

### Pt/Glass/Pt devices

**Figure 8:**
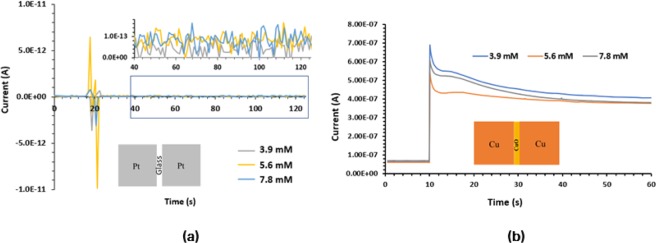
(**a**) A chart showing current levels as a function of time for samples having known concentrations of glucose across previously unused (**a**) Pt/Glass/Pt devices, (**b**) Cu/CuO/Cu devices.

To confirm the role of the CuO layer deposited underneath and between the platinum electrodes in MOMSense devices, 3 mm $\times$ 3 mm Pt electrodes are deposited directly on the glass substrate, as illustrated in the inset of Fig. 8(a). Three fresh devices from the aforementioned system are tested with 3.9 mM, 5.6 mM and 7.8 mM glucose concentrations. The same testing procedure described and followed for the MOMSense devices is used for this investigation. Figure 8(a) shows a random small jump in the electric current level when a drop of solution is applied, indicating no sensing ability to the applied glucose.

### Cu/CuO/Cu devices

In this structure, the Pt electrodes in the MOMSense device are replaced by Cu electrodes to investigate the sensitivity of the device in the absence of platinum. This is realized by depositing 3 mm $\times$ 3 mm Cu electrodes on the CuO layer synthesized using the same process described for MOMSense devices and detailed in the Methods section. As presented in Fig. 8(b), there is no trend to relate the increase in the current passing through the device to the glucose concentration in the added drop. This confirms the role of Pt electrodes that act as catalytic electrodes that easily distinguish the number of electron transfers and consequently result in an electron flow that is proportional to the number of existing glucose molecules^67^.

### Glucose oxidation in CuO system

Copper oxide is well documented as a multiplex electrochemical catalyst in an aqueous medium due to the various oxidized/hydroxylated species that can be present within the neutral to alkaline pH range, depending on the applied potential^68^. A widely accepted nonenzymatic mechanism associates the electro-oxidation of glucose with the presence of the redox active couple Cu^2+^/Cu^3+^ in alkaline conditions (e.g., pH 11–13) in the form of CuO/CuO(OH) species^3^. Accordingly, the oxidation of glucose has been widely explained as per the following two-step process:

First, a half-oxidation reaction of Cu^2+^ to Cu^3+^ occurs under a sufficient voltage supply:

3 $${\rm{CuO}}+{{\rm{OH}}}^{-}\to {\rm{CuO}}({\rm{OH}})+{{\rm{e}}}^{-}$$

Second, a nonenzymatic oxidation-reduction reaction between the formed Cu^III^ oxyhydroxide species and the adsorbed glucose takes place, allowing for further regeneration of CuO species:

4 $$2{\rm{CuO}}({\rm{OH}})+{\rm{glucose}}\to 2{\rm{CuO}}+{\rm{gluconolactone}}+{{\rm{H}}}_{2}{\rm{O}}$$

In addition to being widely accepted for alkaline conditions, a recent work^57^ also claimed this mechanism for establishing glucose oxidation on graphene-modified CuO particles in neutral pH.

On the other hand, a thorough analytical study of the electrochemical CuO system by Barragan *et al*.^69^ pinpointed several controversies of the widely accepted mechanism above to justify a new hypothesis for the electrocatalytic behavior of CuO that claims little to no role of Cu^3+^ species in the electro-oxidation process of glucose. Barragan *et al*. attributed the electron transfer process to the synergistic role between the adsorbed hydroxide ions and the semiconductive behavior of the CuO system that involve ion-pairing and partial charge transfer models rather than direct involvement of Cu^3+^ ions.

**Figure 9:**
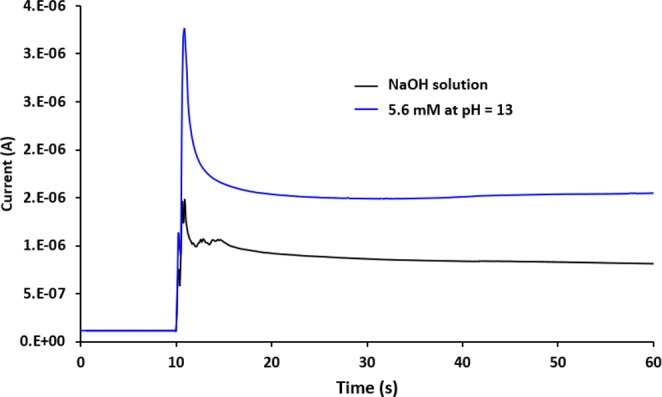
A chart showing current levels as a function of time for fresh MOMSense device tested with glucose concentration of 5.6 mM at pH = 13, under the application of 1 V. Black is NaOH solution.

As for MOMSense devices, some initial experiments (see Fig. 9) are carried out with our devices under alkaline conditions (pH = 13). These results show that a glucose sensing signature with enhanced sensitivity can be established for an increased pH level, where the ratio between the responses of the blank and the glucose sample is enhanced from 1.1 to 1.9 for a pH = 7 and pH = 13, respectively. This indicates that some of the hypotheses reported in the literature can still be applicable, and it also corroborates the electrocatalytic behavior of CuO. We believe that other redox active couples, such as Cu^+^/Cu^2+^, could be highly involved under neutral conditions. In fact, the involvement of the cupric ions Cu^2+^ (i.e., Cu(OH)_2_ and CuO species) in the electrochemical oxidation of carbohydrates is a well-known metabolic pathway, which is also the basis of several biochemical tests for glucose sensing, including Fehling’s test and Benedict’s test^70,71^. However, explaining the mechanism at pH = 7 with the novel MOM structure reported in this work requires further study of the fabricated CuO layer to identify the exact nature of the electrochemical reactions taking place.

## Discussion

We successfully presented the design, fabrication and testing of an efficient nonenzymatic biomedical sensor that is capable of detecting different glucose concentrations ranging from 2.2 mM – 10.0 mM. It was demonstrated that the novel planar Pt/CuO/Pt structure enables the nonenzymatic sensing mechanism. The MOMSense device exhibits a synergistic role for the interfaces between the Pt electrodes and the CuO surface to act as electrocatalysts and consequently facilitate the glucose oxidation needed for glucose detection in the absence of GDH or GOx. The role of the CuO layer and Pt electrodes in the sensing process was demonstrated through fabricating and testing Pt/Glass/Pt and Cu/CuO/Cu structures. These results confirm the synergistic contribution of the Pt electrodes attached to CuO in MOMSense devices. CuO is reported as a promising material to be deployed in NEG sensors. It can perform glucose oxidation on modified CuO-based electrodes in an alkaline solution^34,72–74^. As our goal in this work is to perform glucose testing at a neutral pH, the fabricated Cu/CuO/Cu devices presented in the preceding section are incapable of differentiating glucose concentrations. On the other hand, the Pt electrodes used in MOMSense devices enable the glucose oxidation to take place in neutral solution. The cyclic voltammetry reported in^62^ for Pt electrodes in the presence of glucose at a pH of 7 showed three different oxidation peaks that reflect the electrochemically oxidized glucose at a platinum electrode. However, using Pt electrodes solely in glucose detection has been limited due to the many drawbacks of the material^15,62,65^. The sensing mechanism associated with MOMSense devices fabricated and presented in this paper generally provides new perspectives on the design and testing approaches for biomedical sensors and for glucose sensing specifically. Furthermore, the presented properties of MOMSense devices are in line with the requirements for a viable nonenzymatic glucose sensor^65^ in terms of sensitivity, stability, accuracy, ability to meet the ISO standard (International Organization for Standardization), no oxygen dependency, low cost and ease of fabrication. Evaluating the combined detection of MOMSense devices with µPAD using actual blood samples is beyond the scope of current work and is considered as a future work.

## Methods

### Device fabrication

**Figure 10:**
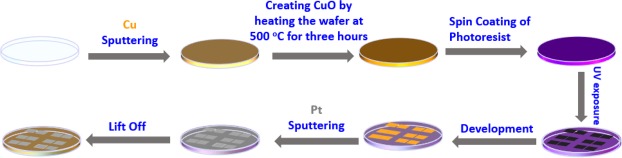
Diagram shows the steps followed to fabricate MOMSense devices.

A low-cost standard photolithography fabrication process is followed in fabricating the MOMSense devices. As illustrated in Fig. 10, 99.9% pure Cu is sputtered on a 4″ Borofloat glass wafer using a Q300T T coating tool by Quorum Technologies. To form the CuO layer, the wafer is heated at 500 °C on a hot plate for three hours. After cooling to room temperature, the lithography step is performed by spin coating 1.4 µm thick MICROPOSIT™ S1813™ positive photoresist. Prior to photoresist deposition, an HMDS primer is used to improve adhesion. A UV exposure system (KLOE 650) is used to pattern the photoresist layer on the wafer, followed by a one-minute development step using an appropriate developer. Next, 99.99% pure Pt is sputtered onto the wafer. Finally, the photoresist layer is lifted off using acetone to produce the final wafer presented in Fig. 3(a).

### Device Characterization

The cross section of a sample MOMSense device is inspected using high-resolution scanning electron microscopy (FEI Nova NanoSEM 650). A Keithley 4200-SCS Parametric Analyzer (Tektronix) is used to perform an amperometric test using voltage pulse mode. The prepared devices are mounted on a probe station and are electrically tested by applying one volt across the Pt electrodes. The compliance current (cc) is set to the instrumental maximum level (i.e., 0.1 A).
